# Direct-Writable and Thermally One-Step Curable “Water-Stained” Epoxy Composite Inks

**DOI:** 10.3390/polym14194191

**Published:** 2022-10-06

**Authors:** Suyeon Kim, Jeewon Yang, Jieun Kim, Seoung Young Ryu, Hanbin Cho, Yern Seung Kim, Joohyung Lee

**Affiliations:** 1 Department of Chemical Engineering, Myongji University, 116 Myongji-ro, Cheoin-gu, Yongin 17058, Gyeonggi-do, Korea; 2 Advanced Materials Company, LG Chem, 70 Magokjungang 10-ro, Gangseo-gu, Seoul 07795, Korea

**Keywords:** epoxy, composite, direct ink writing, 3D printing, thermal curing, computer vision

## Abstract

In this study, a simple method for preparing direct-writable and thermally one-step curable epoxy composite inks was proposed. Specifically, colloidal inks containing a mixture of ordinary epoxy resin and anhydride-type hardener with the suspended alumina microplates, as exemplary fillers, are “stained” with small amounts of water. This increases the elasticity of the ink via the interparticle capillary attraction and promotes curing of the epoxy matrix in low-temperature ranges, causing the three-dimensional (3D) printed ink to avoid structural disruption during one-step thermal curing without the tedious pre-curing step. The proposed mechanisms for the shape retention of thermally cured water-stained inks were discussed with thorough analyses using shear rheometry, DSC, FTIR, and SEM. Results of the computer-vision numerical analysis of the SEM images reveal that the particles in water-stained inks are oriented more in the vertical direction than those in water-free samples, corroborating the proposed mechanisms. The suggested concept is extremely simple and does not require any additional cost to the one required for the preparation of the common epoxy–filler composites, which is thus expected to be well-exploited in various applications where 3D printing of epoxy-based formulations is necessary.

## 1. Introduction

Epoxy is one of the most widely used thermosetting polymers in a wide variety of applications because of its excellent mechanical strength and thermal and chemical stability [1,2,3,4,5,6,7,8,9,10,11]. Recently, several approaches for the direct ink writing (DIW) of epoxy-based materials have been introduced [12,13,14,15]. DIW is a useful technique that enables the facile manufacture of highly complicated [16,17] or lightweight [12,18] three-dimensional (3D) architectures in a customizable manner at a high resolution. One promising approach for the DIW of epoxy is to use an epoxy-based colloidal suspension of filler particles with adequate rheological properties [12]. This can be prepared by dispersing solid filler particles in an uncured liquid epoxy matrix at high particle loadings, which vitrifies the matrix and converts the highly flowable epoxy base ink into a viscoelastic filament that is extrudable under ambient conditions.

Successful thermal curing of the printed structure of the as-prepared epoxy ink, while maintaining the original printed shape, requires additional consideration. Even if the rheological strength of the colloidal epoxy ink is sufficiently high (attributed to the high particle loading), the sample viscosity rapidly decreases during the curing process at high temperatures, which often leads to the complete disintegration of the printed structure. To overcome this issue, Compton and Lewis [12] implemented a two-step curing process in which the printed colloidal epoxy ink was pre-cured at a relatively mild temperature (100 °C) for a long time (15 h), then additionally cured at a much higher temperature (220 °C) for a shorter time (2 h). Similarly, Shi et al. [13] pre-cross-linked nanoclay-dispersed epoxy resins at a curing temperature of 130 °C for a short time (30 min), which made the resin 3D-printable at a moderately high temperature of 70 °C. The 3D-printed structures of the pre-cross-linked resin were subsequently pre- and fully cured at 60 °C and 130 °C for 20 h and 6 h, respectively. Chen et al. [14] proposed a combined process of light-assisted and thermal curing substeps to avoid the tedious and complicated thermal processes, where the mixed formulations of photo- and thermally curable resins as well as the reinforcing silica fillers were required, and UV irradiation had to be implemented in the manufacture of each layer of the 3D structure. These previous studies clearly revealed that curing of the 3D-printed structures of colloidal epoxy inks is not a simple process. A new method that enables more facile curing of the epoxy ink with the shape of the 3D-printed structure maintained during the entire process is desirable to improve process efficiency and cost-effectiveness.

In this study, we demonstrated directly writable and thermally one-step curable “water-stained” epoxy inks with suspended alumina microplates. Selection of alumina as the fillers was made because of their high potential in various applications including thermal management. We show that the simple addition of minute amounts of pristine water to a mixture of commonly used epoxy base resin and anhydride-type hardener, without any specially designed proprietary curing agent, can improve the 3D printability of the resulting ink, possibly owing to the capillary-force-induced attraction of the dispersed oxide particles. More importantly, it can achieve shape retention of the printed ink during the post thermal curing process by initiating the curing reaction from an earlier temperature than the target curing temperature. The proposed method is extremely simple and economical, which is thus expected to be well-exploited in various applications where 3D printing of epoxy-based formulations is necessary.

## 2. Materials and Methods

### 2.1. Materials

Plate-shaped alpha-alumina (α-Al_2_O_3_) microparticles, with the maximum Feret basal-plane diameter of 4.09 ± 1.73 μm and thickness of 1.18 ± 0.25 μm, were purchased from PACE Technologies (India). Bisphenol A diglycidyl ether (BADGE), hexahydro-4-methylphthalic anhydride (HMPA), and 2-ehtyl-4-methylimidazole (EMI) were purchased from Sigma-Aldrich (St. Louis, MO, USA). Deionized (DI) water with a resistivity of 18.2 MΩ·cm (Direct-Q^®^ Water Purification System) was used.

### 2.2. Sample Preparation

To prepare the epoxy–alumina composite ink, desired amounts of α-Al_2_O_3_ microplates (10–30 vol.%) were mixed with an equimolar mixture of BADGE and HMPA using a lab dissolver at 500 rpm for 1 min, to which EMI (0.5 vol.%) was added and further mixed for 1 min. For the water-stained inks, small amounts of DI water (1–3 vol.%) were subsequently added to the premixture of α-Al_2_O_3_, BADGE, HMPA, and EMI, which was mixed at 2000 rpm for 2 min. The printed inks on the glass slides were placed in a muffle furnace, and the temperature was raised at a rate of 10 °C·min^−1^ to reach 150 °C, at which the sample was cured for 90 min, followed by slow cooling at room temperature.

### 2.3. Direct Ink Writing (DIW)

For instant printability evaluation, a sample ink was charged into a 5 mL syringe with a nozzle diameter of 2 mm and manually extruded on a glass slide. For instrument-assisted demonstration of the direct writing of the water-stained composite ink, a commercial DIW system (Ultimaker 2+, Ultimaker, The Netherlands), with a printing diameter of 1.54 mm, and Cura 4.1.0 software were used.

### 2.4. Characterization

A HAAKE MARS-40 rheometer (Thermo Fisher Scientific, Waltham, MA, USA) with parallel plates (35 mm in diameter and 1 mm in gap) was used to conduct rheological measurements on the uncured epoxy base and epoxy–alumina composite inks with and without added water. Rotational viscosity measurements were performed within a shear rate range of 0.1–100 s^−1^. Oscillatory viscoelasticity measurements were performed using a shear stress ramp. The investigated shear stress ranges for various formulations were varied between 0.1 and 1000 Pa to find linear viscoelastic regions. The advancing contact angle of a water droplet on the surface of a commercial α-Al_2_O_3_ macroplate (Aluminum Oxide 60, for thin-layer chromatography, Merck, Darmstadt, Germany) immersed in an uncured equimolar mixture of BADGE and HMPA was measured using a Phoenix-MT goniometer (SEO, Suwon, Korea). An IRTracer-100 (Shimadzu, Kyoto, Japan) Fourier transform infrared (FTIR) spectrometer was used to analyze the chemical groups of the cured and uncured composite inks. A Q20 DSC25 (TA Instruments, New Castle, DE, USA) differential scanning calorimetry (DSC) instrument was used to identify the temperature ranges where the curing reaction occurred for the inks with various formulations in the temperature range of 25–200 °C under a nitrogen atmosphere at a heating rate of 10 °C·min^−1^. An EM-30 (COXEM, Daejeon, Korea) scanning electron microscopy (SEM) instrument was used to analyze the microstructures of the cured epoxy–alumina composites.

## 3. Results and Discussion

**Figure 1:**
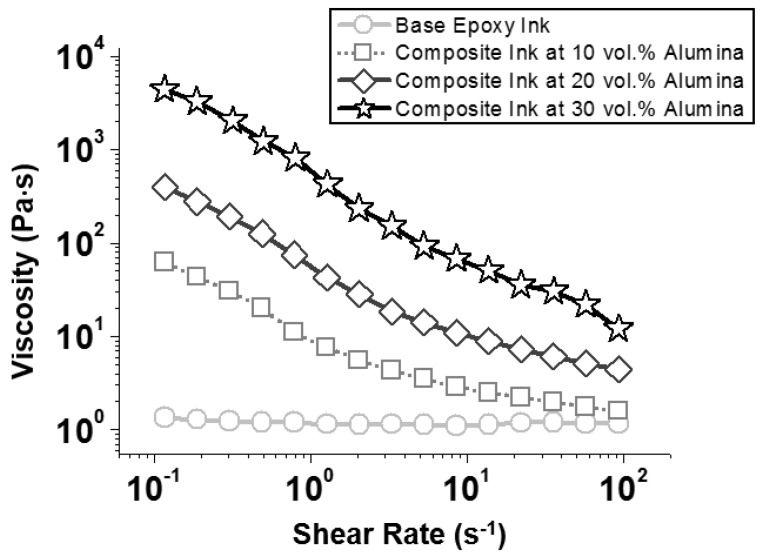
Viscosities of uncured base and alumina-suspended (10–30 vol.%) epoxy inks, measured using rotational viscometry within applied shear rate range between 0.1 and 100 s^−1^.

Epoxy–alumina composite inks were prepared by dispersing the desired amounts (10–30 vol.%) of plate-shaped α-Al_2_O_3_ microparticles (maximum Feret basal-plane diameter of 4.09 ± 1.73 μm and thickness of 1.18 ± 0.25 μm, Figure S1) in a liquid-state mixture of an epoxy resin (BADGE) and an anhydride-type hardener (HMPA) with a 1:1 molar ratio, followed by adding small amounts (0.5 vol.%) of an imidazole-based curing catalyst (EMI). Without added alumina particles, the base epoxy ink had a nearly constant viscosity of ~1.1 Pa·s under a wide range of applied shear rates in rotational viscometry (Figure 1), showing Newtonian behavior similar to conventional liquid epoxy resins [12].

**Figure 2:**
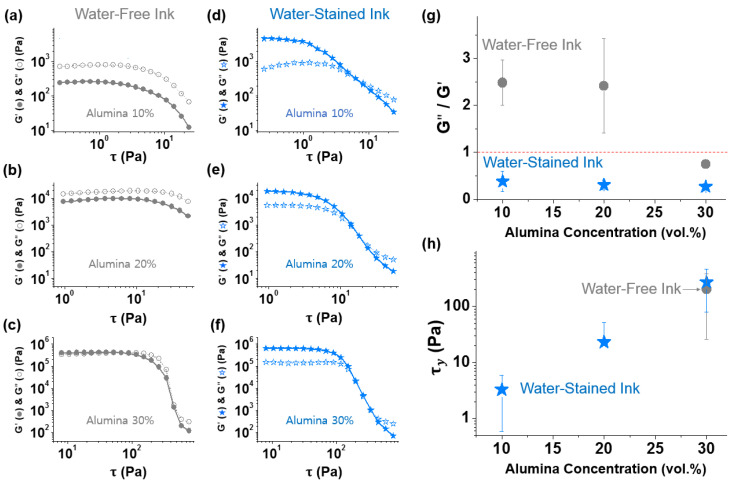
Representative elastic ($G^{\prime }$, filled) and viscous ($G^{\prime \prime }$, empty) moduli of water-free epoxy–alumina inks (gray circles) with alumina concentration of (**a**) 10, (**b**) 20, and (**c**) 30 vol.% and those of water-stained inks (blue stars) with alumina concentration of (**d**) 10, (**e**) 20, and (**f**) 30 vol.% and a fixed water concentration of 3 vol.%, as functions of applied shear stress (τ) in oscillatory measurements (at 1 Hz) using a stress ramp. (**g**) Initial $G^{\prime \prime }/G^{\prime }$ and (**h**) yield stress (τ_y_) values for water-free (gray circles) and water-stained (blue stars) inks. Data in (**g**) and (**h**) are averaged values for the results from at least three independent measurements, which are described as functions of alumina concentrations.

**Figure 3:**
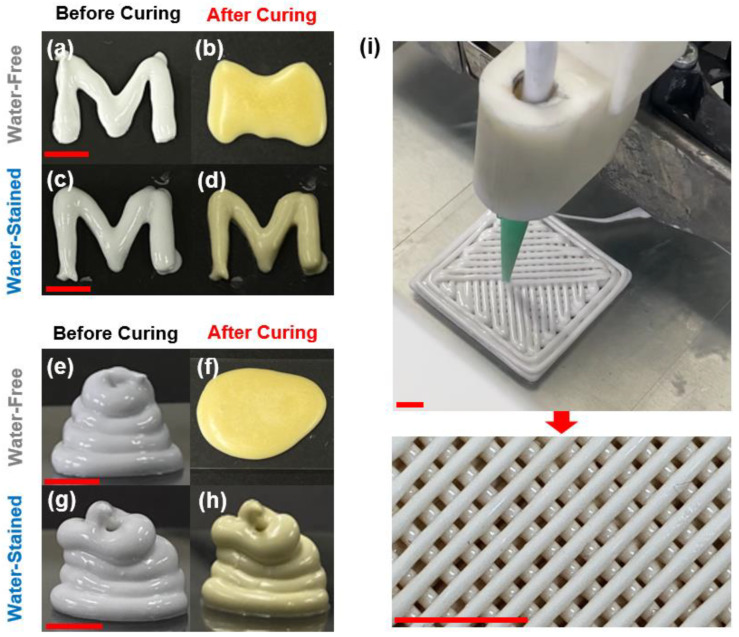
Photographs of hand-extruded letter “M” ((**a**–**d**)) and 3D architectures with arbitrary shapes ((**e**–**h**)), using water-free epoxy–alumina ink before (**a**,**e**) and after (**b**,**f**) thermal curing, and using water-stained ink before (**c**,**g**) and after (**d**,**h**) thermal curing. (**i**) Demonstration of instrument-assisted 3D printing using water-stained ink (**top**) and a magnified photograph of cured product (**bottom**). Fixed alumina concentration of 30 vol.% was used for all samples. For water-stained ink, a fixed water concentration of 3 vol.% was used. Red scale bars are 5 mm.

The addition of solid particles thickened the ink, which was apparent from the significant increases in the measured viscosities with the increase in particle concentration (Figure 1). Furthermore, the elastic ($G^{\prime }$) and viscous ($G^{\prime \prime }$) moduli of the alumina-suspended epoxy inks, which reflect the solid- and liquid-like properties of the samples, respectively [19], systematically increased with increasing particle concentration (Figure 2a–c). The ratio of $G^{\prime \prime }$ to $G^{\prime }$ ($G^{\prime \prime }/G^{\prime }$ or tanδ; see Figure S2) in oscillatory measurements using a stress ramp (at a fixed frequency 1 Hz) decreased with increasing particle concentration (the initial $G^{\prime \prime }/G^{\prime }$ values corresponding to the lowest applied shear stresses in Figure 2a–c, averaged for at least three independent measurements, are summarized in Figure 2g). At the particle concentration of 30 vol.% (Figure 2c; see Figure S3 for the magnified version of Figure 2), the $G^{\prime }$ curve was dominant over $G^{\prime \prime }\;$($G^{\prime \prime }/G^{\prime }<1$) at low shear stresses, indicating vitrification of the ink, attributed to the increased interparticle interaction. As expected, the epoxy–alumina ink with higher rheological strength exhibited higher 3D printability, as illustrated in Figure S4 and Figure 3a,e. However, when these samples were heated to 150 °C at a rate of 10 °C·min^−1^ and cured at that temperature for 90 min, the printed structures were completely disrupted (Figure 3b,f). The curing of samples with a higher alumina loading (40 vol.%), which presumably had higher strength (no rheological characterization was systematically performed because of the difficulty of “fluidic” processing of these samples), similarly resulted in the complete disintegration of the samples under the same conditions (Figure S5).

**Figure 4:**
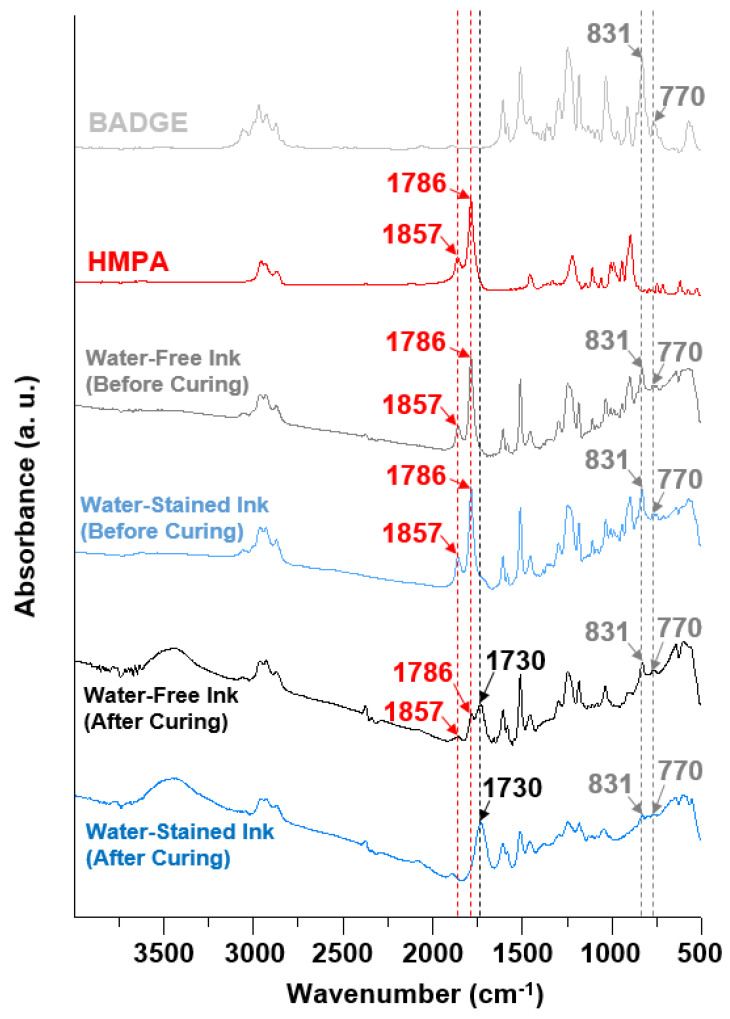
FTIR spectra for BADGE, HMPA, uncured epoxy−alumina inks without and with added water at 3 vol.%, and cured epoxy−alumina inks without and with added water at 3 vol.%. Fixed alumina concentration of 30 vol.% was used for all uncured and cured composite inks.

When a small amount of water (3 vol.%) was added to the aforementioned epoxy–alumina inks, the $G^{\prime \prime }/G^{\prime }$ values at low shear stresses reduced to <1 for all the formulations (Figure 2d–g and Figure S2). Remarkably, the dominance of $G^{\prime }$ over $G^{\prime \prime }$ was observed, even for the sample with the lowest particle concentration (10 vol.%, Figure 2d), which behaved as a weak gel with a yield stress (τ_y_, obtained at the cross-point of the $G^{\prime }$ and $G^{\prime \prime }$ curves) of ~3 Pa, in stark contrast to the water-free ink at the corresponding alumina concentration. The τ_y_ value increased by an order of magnitude when the particle concentration increased by 10 vol.% in the presence of the same amount of added water (Figure 2h). The τ_y_ value of the water-stained ink with the highest particle concentration (30 vol.%) was close to that of the water-free sample at the corresponding particle concentration (Figure 2h) for which the interparticle network formation was assumed. However, the $G^{\prime \prime }/G^{\prime }$ value at low shear stresses for the water-stained ink was significantly lower (approximately threefold; Figure 2g and Figure S2c), indicating that the water-stained ink was more elastic than the water-free ink. Overall, the addition of water made the epoxy–alumina composite ink more “solid-like”. We note that the addition of water in the same amount to the particle-free epoxy base ink did not alter the sample viscosity (Figure S6). This indicates that the contribution of the possible water-induced curing reaction of the epoxy matrix, expected from the small shoulder peak at ~1730 cm^−1^ in the FTIR spectrum [20] of the uncured water-stained composite ink (Figure 4; to be discussed further below), to the sample thickening was likely minor at most. Indeed, the FTIR spectra for the composite inks with and without added water were very close, suggesting that the sample thickening should be attributed to a reason other than the curing of the epoxy matrix.

**Figure 5:**
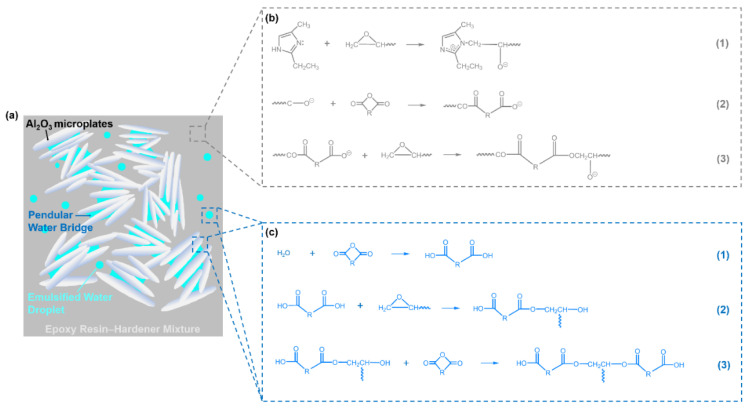
(**a**) Schematic of epoxy–alumina composite ink with added water. Proposed mechanisms of epoxy–anhydride curing reactions initiated by (**b**) EMI [28] and (**c**) water [29].

The enhanced rheological strength of the uncured ink might be attributed to the capillary attraction between the dispersed particles, induced by water, as the “secondary” fluid immiscible with the bulk (epoxy resin–hardener) fluid [21,22,23]. The three-phase contact angle (θ) of water on the alumina surface in the bulk fluid was determined by measuring the advancing contact angle of a water droplet on a commercial alumina macroplate immersed in an uncured liquid mixture of BADGE and HMPA, and using Wenzel’s equation [23,24]:

(1) $${\cos\theta }_{\mathrm{app}}=\mathrm{rcos}\theta$$

where θ_app_ is the measured *apparent* contact angle on the non-ideal surface of an *intrinsic* (or thermodynamic) contact angle of θ and a rugosity factor of r. The θ of ~76° inferred by taking r=1.25 known for the alumina macroplate into account, which is close to that of the pelletized surface of alumina microplates used in the current study [23], suggests that water preferentially wets the alumina surface immersed in the bulk fluid. This forms a *pendular* bridge (θ < 90°), a notion opposite to a *capillary* bridge (θ > 90°), for which the bulk fluid preferentially wets the surfaces [21] and thus exerts an attractive capillary force between the bridged alumina particles (Figure 5a). According to the literature [22,23], the $\tau_{y}$ of a ternary colloidal suspension consisting of the particles and the bulk and (a small amount of) secondary fluids, often referred to as a “capillary” suspension [21], may be described as

(2) $$\tau_{y}=f\left(\phi_{P},{\;N}_{\mathrm{bridge}}\right)\frac{F_{c}}{a^{2}}=f\left(\phi_{P},{\;N}_{\mathrm{bridge}}\right)g\left(V_{\mathrm{bridge}}\right)\frac{2\pi \gamma \cos\theta }{a}$$

where $F_{c}=\;g\left(V_{\mathrm{bridge}}\right)2\pi a\gamma \cos\theta$ is the capillary force, γ is the interfacial tension between the bulk and secondary fluids, a is the radius of the particles, $f\left(\phi_{P},{\;N}_{\mathrm{bridge}}\right)$ is a function of the particle volume fraction ($\phi_{P}$) and the number of secondary fluid bridges per unit volume ($N_{\mathrm{bridge}}$), and $g\left(V_{\mathrm{bridge}}\right)$ is a function of the volume of bridge ($V_{\mathrm{bridge}}$). While the factors $f\left(\phi_{P},{\;N}_{\mathrm{bridge}}\right)$ and $g\left(V_{\mathrm{bridge}}\right)$ strongly depend on the system components and processing conditions, and thus have not been described in a closed form yet, γcosθ may be an important parameter that determines the magnitude of the capillary force. As a control, it was previously shown that the addition of a small amount of water (2 vol.%) to a colloidal suspension comprising a paraffin oil and exactly the same alumina microparticles (25 vol.%) dramatically increased the suspension strength, achieving a τ_y_ of ~2000 Pa [23]. In this pure unreactive nonpolar bulk fluid, the inferred θ was ~46° and the γ was ~34 mN/m, and thus, the γcosθ was ~23 mN/m. This is approximately an order-of-magnitude higher than that for the current system, γcosθ=15×cos(76°)~3, explaining the qualitative difference in the measured sample strength between the two systems ($\tau_{y}\sim$2000 Pa for γcosθ~23 mN/m versus $\tau_{y}<$ 200 Pa for γcosθ~3) at corresponding particle loadings. Although not as strong as in the case of the earlier paraffin oil bulk fluid, we conjecture that the added water, as the particle-wetting fluid in the currently studied ternary formulation, could also promote the attractive capillary force between the dispersed particles in the uncured epoxy medium.

Under the same curing conditions as those used for the water-free samples (150 °C, 90 min), the printed structures of the water-stained alumina–epoxy inks did not undergo disruption, maintaining their original shapes (Figure 3c,d,g–i). It is notable that the τ_y_ value for the water-stained ink at the highest particle loading of 30 vol.% (~200 Pa) is well below those of previously reported 3D-printing colloidal inks with high filler loadings (often >1000 Pa) [18,23,25], including the colloidal epoxy ink formulated with silicone carbide whiskers and milled carbon fibers [12]. This suggests that our proposed water-stained epoxy–alumina ink has a higher rheological processability in terms of the ease of extrusion at a given energy loading [26,27] than previously reported inks, and also exhibits sufficient shape retention for DIW and one-step thermal curing.

**Figure 6:**
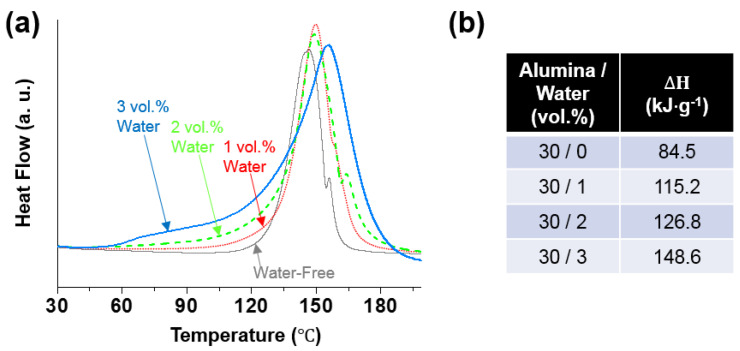
(**a**) DSC curves for uncured water-stained epoxy–alumina inks at fixed alumina concentration of 30 vol.% with various water contents (0−3%). (**b**) Total enthalpies (ΔH in kJ·g^−1^) during heating, obtained from DSC measurements.

To understand the shape retention mechanism of the printed water-stained ink during thermal curing, DSC was performed on samples with various water contents (0–3 vol.%, at a fixed particle concentration of 30 vol.%); the results are summarized in Figure 6. Without the addition of water, the EMI-catalyzed polymerization (Figure 5b) [20,28] occurred in a relatively high-temperature range, inferred from the primary DSC exothermal peak of this sample with an estimated onset (T_onset_) and peak (T_peak_) temperatures of 124.1 and 146.31 °C, respectively. The measured T_peak_ was very close to that of the base epoxy ink (143.03 °C; see Figure S7) with no added alumina particles, which was thus attributed to the polymerization reaction of the matrix. The second exothermic peak at 156.3 °C may be attributed to the un-EMI-catalyzed polymerization, which could be initiated by OH-containing impurities and/or residual moisture [20,29]. During the course of temperature elevation from ambient to curing temperature (150 °C) at a rate of 10 °C·min^−1^, i.e., within 12.5 min, the degree of polymerization was expected to be low. Thus, the heating could accelerate the disruption of the printed structure by simply lowering the viscosity of the uncured matrix fluid. However, when water was added, polymerization appeared to occur in a relatively low-temperature range, as revealed by the increased fronting of the DSC exothermal peaks. This lowering of the reaction temperature might be attributed to the initiation reaction promoted by the added water. This could effectively break the anhydride group of the hardener HMPA at relatively mild temperatures, which produces large amounts of carboxylic acids that were reactive with the epoxide groups of BADGE, thereby facilitating un-EMI-catalyzed polymerization (Figure 6c) [20,29]. Despite the possible (endothermic) evaporation of added water during heating, larger heat releases were inferred for the samples with higher amounts of water by integrating the DSC peaks of the four different samples (Figure 6b), which corroborates that the reaction occurred to a higher degree as more water was added to the samples. The characteristic FTIR peaks at 1786 and 1857 cm^−1^, attributed to the anhydride group of the hardener HMPA, and those at 770 and 831 cm^−1^, attributed to the epoxide group of BADGE, almost disappeared in the spectrum of the cured product of water-stained ink, whereas such peaks were still distinct in the spectrum of the cured water-free ink (Figure 4). (Note: the intensities of these peaks were close for both the water-free and water-stained samples before curing, indicating that the reaction mostly proceeded during thermal curing at elevated temperatures). Furthermore, the new peak at 1730 cm^−1^, attributed to the ester group owing to the polymerization of BADGE and HMPA [20], was much more distinct for the cured water-stained ink than for the water-free sample. Therefore, the FTIR analyses were consistent with the insights gained from the DSC results, suggesting that the addition of a small amount of water to the epoxy–alumina ink resulted in a higher degree of polymerization of the epoxy matrix during thermal curing. We suggest that this water-initiated polymerization, which started at an earlier temperature than the curing temperature, could support the shape retention of the printed structures during curing.

**Figure 7:**
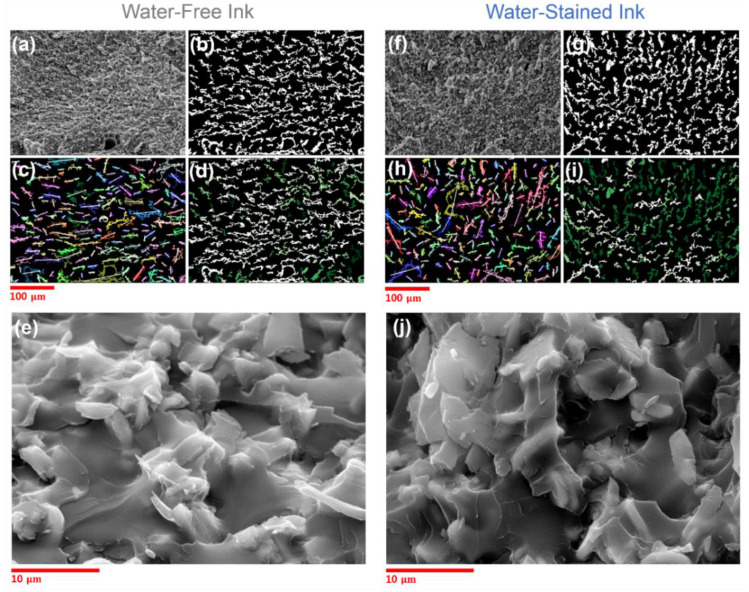
Raw and processed SEM images of cured water-free (**a**–**e**) and water-stained (3 vol.%, **f**–**j**) inks. Raw images at different magnifications are (**a**,**e**,**f**,**j**). Representative edge segments from raw images (**a**,**f**), detected by computer vision, are represented in (**b**) and (**g**), respectively. First eigenvectors for exported edge segments in (**b**) and (**g**) are represented in (**c**) and (**h**), respectively. CS values of first eigenvectors in (**c**) and (**h**) are represented as colors, with green (close to 1, vertical) and white (close to 0, horizontal), in (**d**) and (**i**), respectively.

SEM was used to analyze the microstructures of the cured products. Figure 7a,f show the SEM images of the cross-sections of the cured water-free (30 vol.% alumina) and water-stained (30 vol.% alumina and 3 vol.% water) inks, respectively, at a magnification of ×500. For quantitative analysis of the orientation of the particles, Python 3.7 OpenCV was employed (detailed descriptions of the image analysis procedure are provided in the Supporting Information). First, the representative *edges*, which might be of alumina particles or cured polymers, shown in Figure 7a,f were detected by a computer-vision numerical analysis and are illustrated in white in Figure 7b,g, respectively. The first eigenvectors, which represent the main long axis vectors, for the covariance matrix of the x and y coordinates of the contour of the exported edge segments were then calculated, which are illustrated by white arrows in Figure 7c,h to describe the orientations of the edge segments in the respective samples. Finally, a cosine similarity (CS) is defined as the absolute value of the dot product of unit orientation ($\vec{v}$) and thickness (or vertical) direction vector ($\vec{t}$, (0, 1)):

(3) $$\mathrm{CS}=\left|\vec{v}\cdot \vec{t}\right|$$

**Figure 8:**
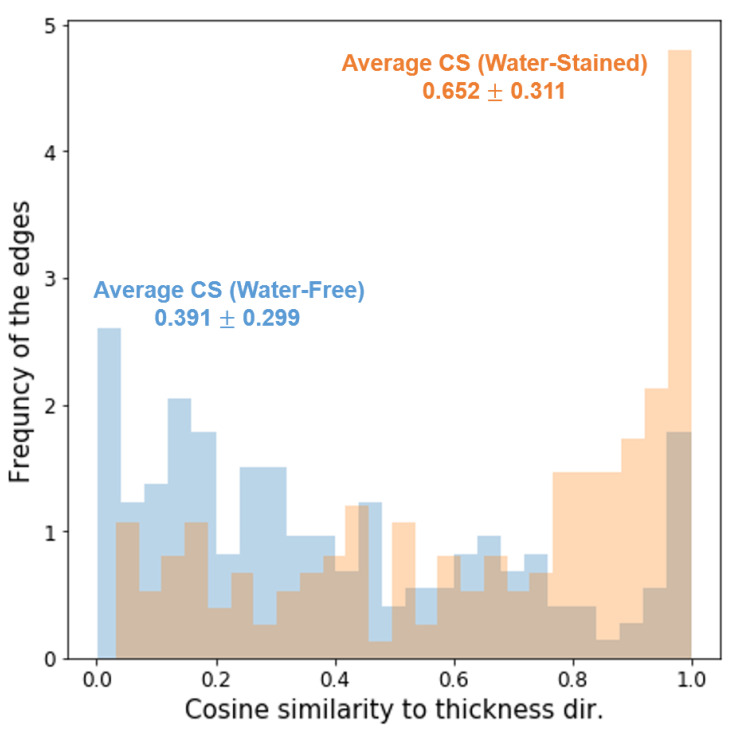
Cosine similarity distributions for all detected edge segments from SEM images in Figure 7a,f.

The CS values were calculated for each edge segment. An edge segment with a CS of 1 (and 0) corresponds to one parallel (and perpendicular) to the sample thickness direction, thus being aligned in the vertical (and horizontal) direction. The distributions of the calculated CS values for all the detected edge segments are illustrated in Figure 8. In addition, the edge segments shown in Figure 7b,g are described using different colors in Figure 7d,i, respectively, where the edge segments with CS values close to 1 and 0 are denoted by green and white, respectively. The results of this computer vision-based analysis clearly suggest that the particulate species in the cured inks without and with water were generally oriented in the horizontal and vertical (or less horizontal) directions, respectively. Given the plate shape of the alumina particles with high aspect ratios, it is not surprising that the particles, which gravitationally sedimented because of the high density during the curing process, stacked in the horizontal direction [30], which is distinct for the cured ink without water but not for that with added water. When the water-stained ink was cured, the particles that were bridged by the capillary-attraction-inducing water (Figure 5a) could be kinetically arrested in the matrix that started to be cured at an earlier temperature than the target before they sedimented and stacked in the horizontal direction. Notably, the particles that were arrested in the vertical direction as well as the vertically oriented polymer segments bridging the particles were observed in the SEM images of the cured water-stained sample at higher magnification (Figure 7j, not distinct for the water-free sample in Figure 7e), the formation of which could start when the water in the interparticle capillary region attacked the neighboring anhydrides from the matrix (Figure 5c). Overall, the results of the analysis of the microstructures of the cured inks strongly support the proposed mechanism for the shape retention of water-stained inks during thermal curing, as shown in Figure 5.

## 4. Conclusions

We demonstrated that the addition of small amounts of pristine water to a colloidal epoxy suspension with ordinary formulations, consisting of a mixture of well-known epoxy resin (BADGE) and an anhydride-type hardener (HMPA) with thermally conductive alumina microplates, facilitates the manufacture of direct-writable and thermally one-step curable composite inks, without using any specially designed curing agent of proprietary composition. The added water, which possessed a three-phase contact angle of <90° on the alumina surface immersed in the uncured epoxy medium, increased the elastic properties of the alumina suspension at room temperature, which was attributed to the interparticle capillary attraction induced by water. Furthermore, the added water promoted the curing of the epoxy matrix by inducing the reaction in earlier temperature ranges than the target curing temperature (150 °C), which helped avoid the structural disruption of the printed ink during one-step thermal curing without the tedious pre-curing step. The proposed mechanisms for the shape retention of thermally cured water-stained inks were discussed with thorough analyses using shear rheometry, DSC, FTIR, and SEM. The results of the computer-vision numerical analysis of the SEM images resulted in a higher CS value for water-stained inks, indicating that the particles were more vertically oriented than those in water-free samples, which corroborated the proposed mechanisms in a quantitative fashion. The proposed concept in this study is extremely simple and does not require any additional cost to the one required for the preparation of the common epoxy–filler composites, which is expected to be extendable for the manufacture of various epoxy-based formulations with fillers other than alumina [1,2,3,6,7,9,10,31,32,33], and the products will be useful in a variety of applications where 3D printing of epoxy composite materials is necessary.

## Data Availability

The data presented in this study are available on request from the corresponding author.

## Supplementary Materials

The following supporting information can be downloaded at: https://www.mdpi.com/article/10.3390/polym14194191/s1, Figure S1: SEM images of the α-Al_2_O_3_ microparticles used in the present study, obtained at a magnification of (a) ×1000 and (b) ×3000, Figure S2: Tanδ curves of the water-free (grey dashed lines) and water-stained (3 vol.%; blue solid lines) inks with an alumina concentration of (a) 10, (b) 20, and (c) 30 vol.%, represented as functions of the applied shear stress (τ) in oscillatory measurements (at 1 Hz) using a stress ramp, Figure S3: Elastic ($G^{\prime }$, filled) and viscous ($G^{\prime \prime }$, empty) moduli of the water-free epoxy/alumina ink with an alumina concentration of 30 vol.%. This data is an magnified version of the Figure 1c in the main text. The dashed vertical line passes the cross-point of the $G^{\prime }$ and $G^{\prime \prime }$ curves, indicating the yield point of the sample, Figure S4: Photographs on the hand-extruded letter “M”, using water-free epoxy/alumina inks with the particle concentration of (a) 10 and (b) 20 vol.%. The height of the glass substrate is 2.5 cm, Figure S5: Photographs on an arbitrary-shaped 3D architecture, using the water-free epoxy/alumina ink at the particle concentration of 40 vol.% (a) before (side-view) and (b) after thermal curing (top-view), Figure S6: Viscosities of the uncured base epoxy inks without (grey squares) and with (blue stars) added water (3 vol.%), measured using rotational viscometry within the applied shear rate range between 0.1 and 100 s^−1^, Figure S7: DSC curves for the uncured base epoxy ink (grey) and water-free epoxy/alumina ink (30 vol.% alumina), and detailed procedure for computer-vision numerical analysis.
