# Supplementary material for: Direct-Writable and Thermally One-Step Curable “Water-Stained” Epoxy Composite Inks
Source: Polymers (Basel). 2022 Oct 6;14(19):4191. doi: 10.3390/polym14194191 (PMC9573494; doi:10.3390/polym14194191)
Supplement: Supplementary file 1 [file polymers-14-04191-s001.zip › polymers-1938992-supplementary.pdf]

Supporting Information: Direct-writable and thermally one-step curable “water-stained” epoxy composite inks

Suyeon Kim,[†,1] Jeewon Yang,[†,1] Jieun Kim,[1] Seoung Young Ryu,[1] Hanbin Cho,[1] Yern Seung Kim[*,2] and Joohyung Lee[*,1]

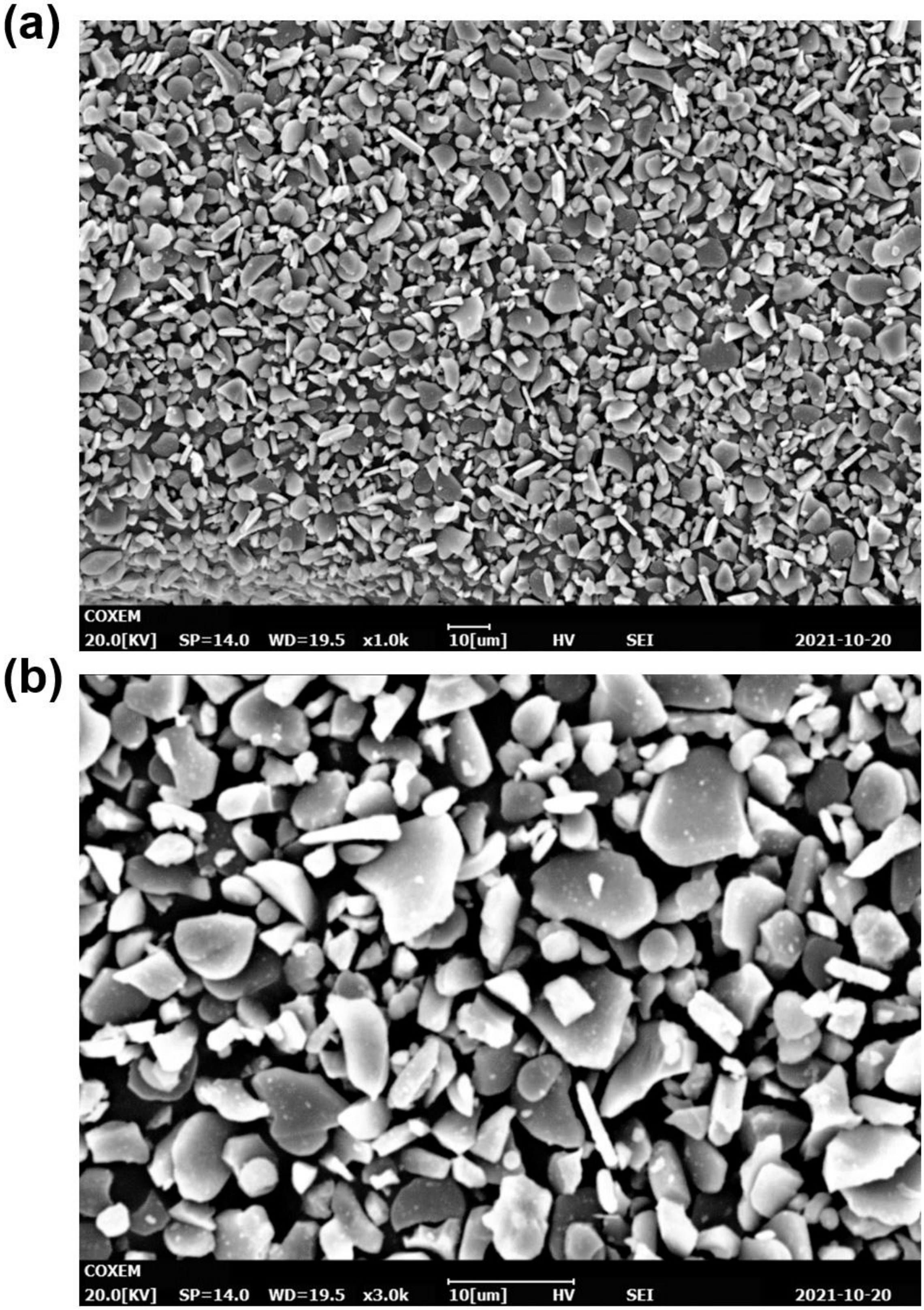

Figure S1. SEM images of the α-Al_2O_3 microparticles used in the present study, obtained at a magnification of (a) ×1000 and (b) ×3000.

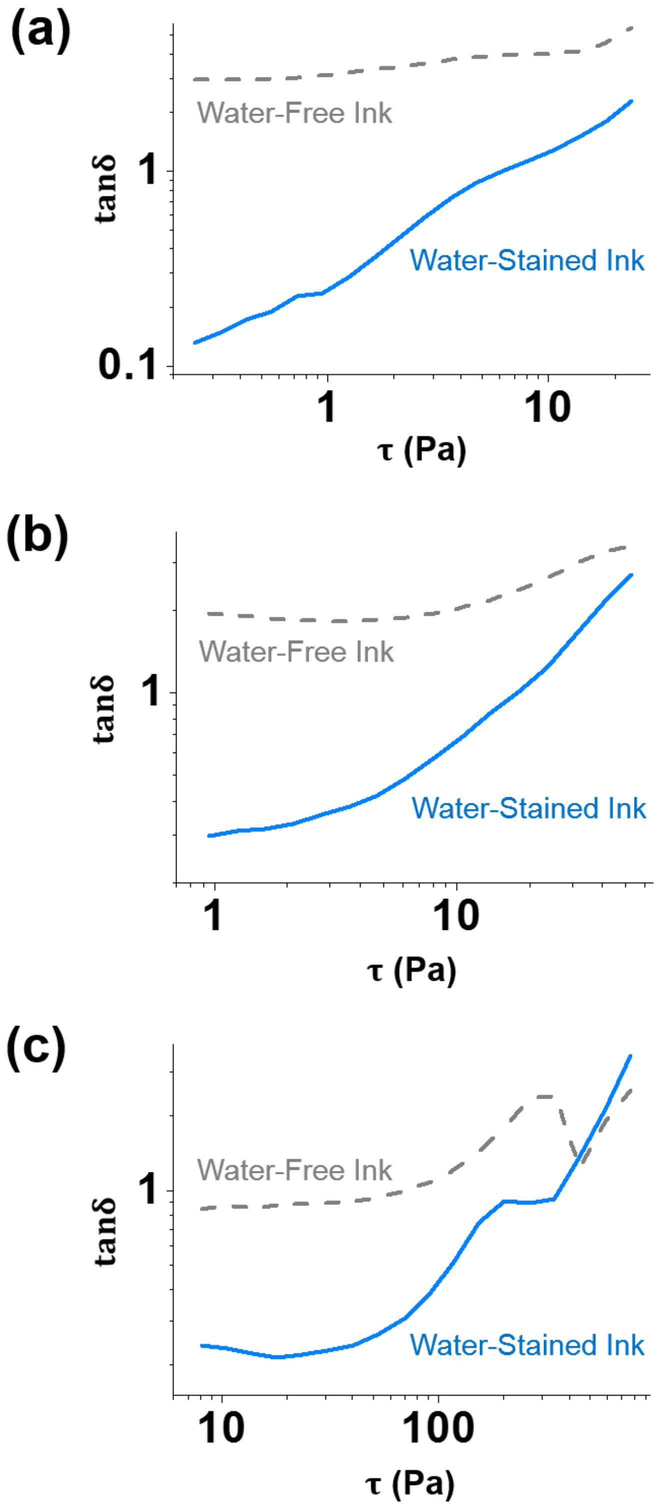

Figure S2. Tanδ curves of the water-free (grey dashed lines) and water-stained (3 vol.%; blue solid lines) inks with an alumina concentration of (a) 10, (b) 20, and (c) 30 vol.%, represented as functions of the applied shear stress (τ) in oscillatory measurements (at 1 Hz) using a stress ramp.

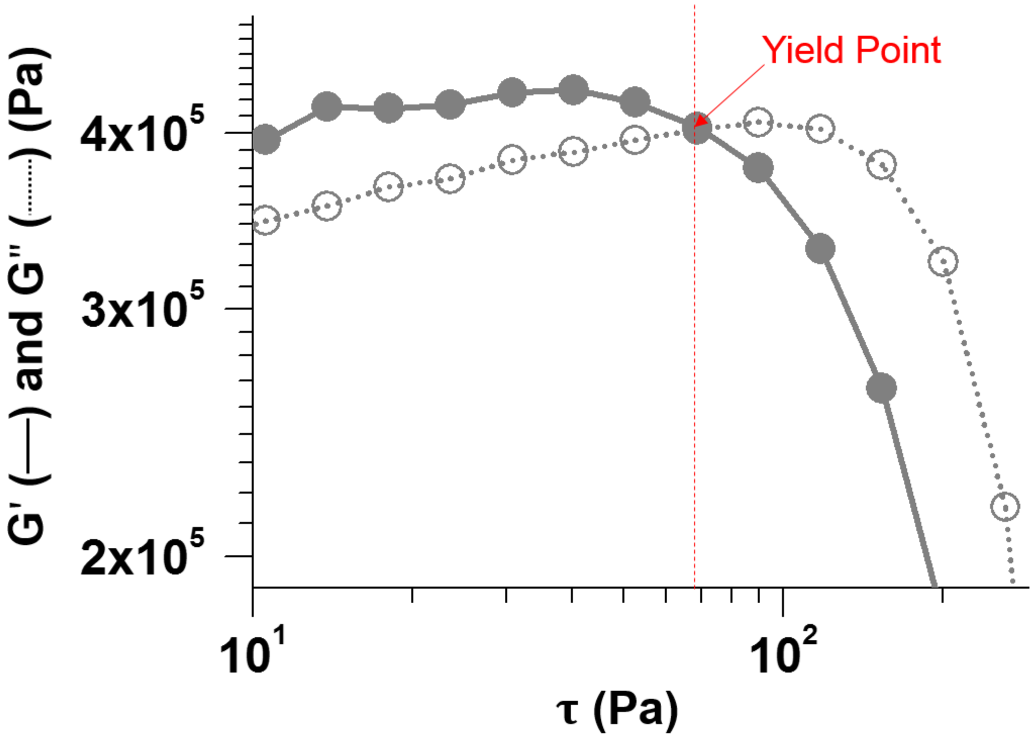

Figure S3. Elastic (G', filled) and viscous (G", empty) moduli of the water-free epoxy/alumina ink with an alumina concentration of 30 vol.%. This data is an magnified version of the Figure 1c in the main text. The dashed vertical line passes the cross-point of the G' and G" curves, indicating the yield point of the sample.

(a)

(b)

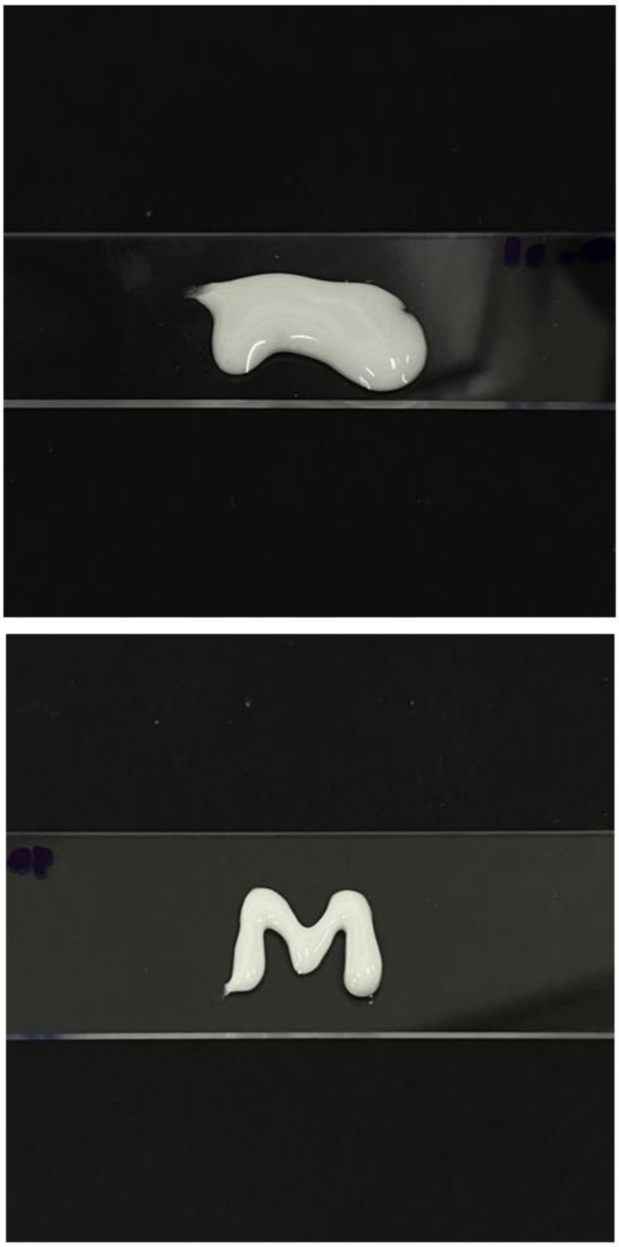

Figure S4. Photographs on the hand-extruded letter "M", using water-free epoxy/alumina inks with the particle concentration of (a) 10 and (b) 20 vol.%. The height of the glass substrate is 2.5 cm.

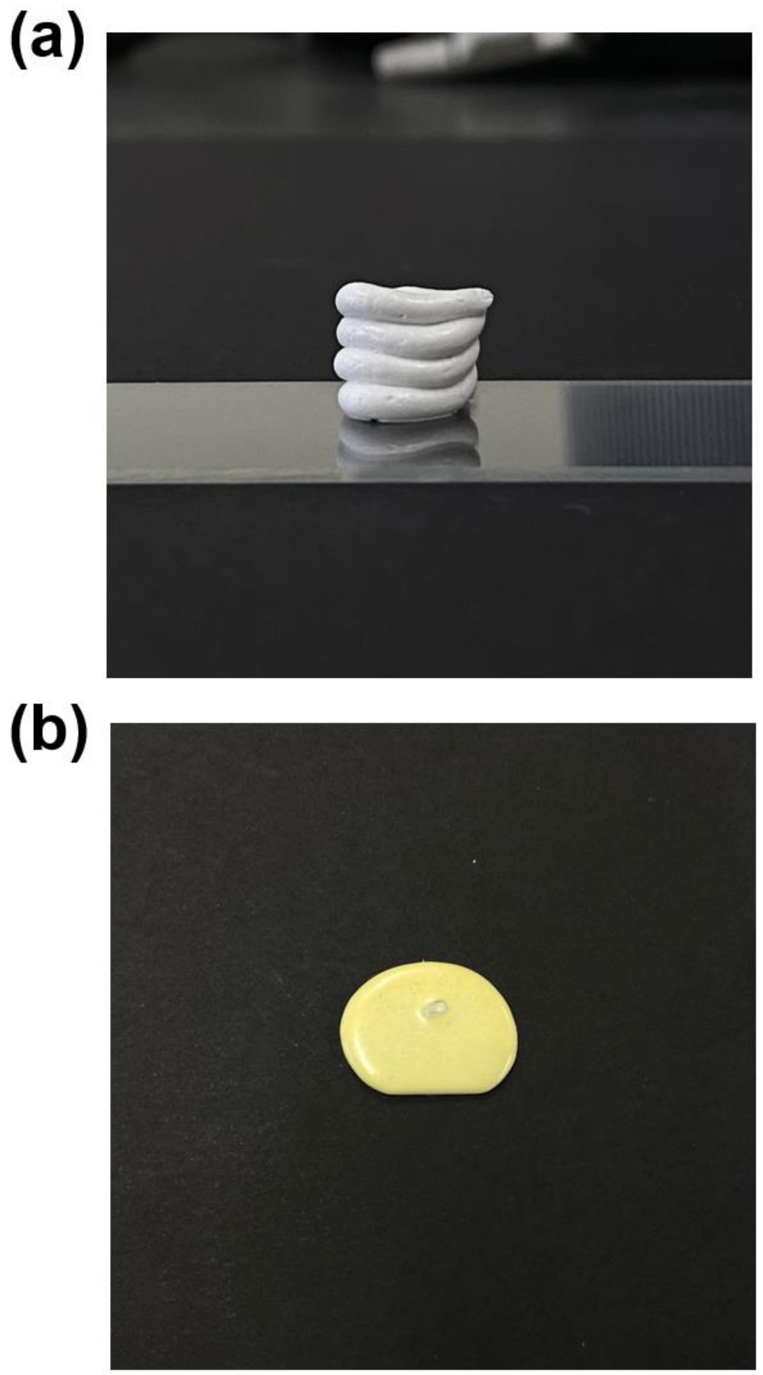

Figure S5. Photographs on an arbitrary-shaped 3D architecture, using the water-free epoxy/alumina ink at the particle concentration of 40 vol.% (a) before (side-view) and (b) after thermal curing (top-view).

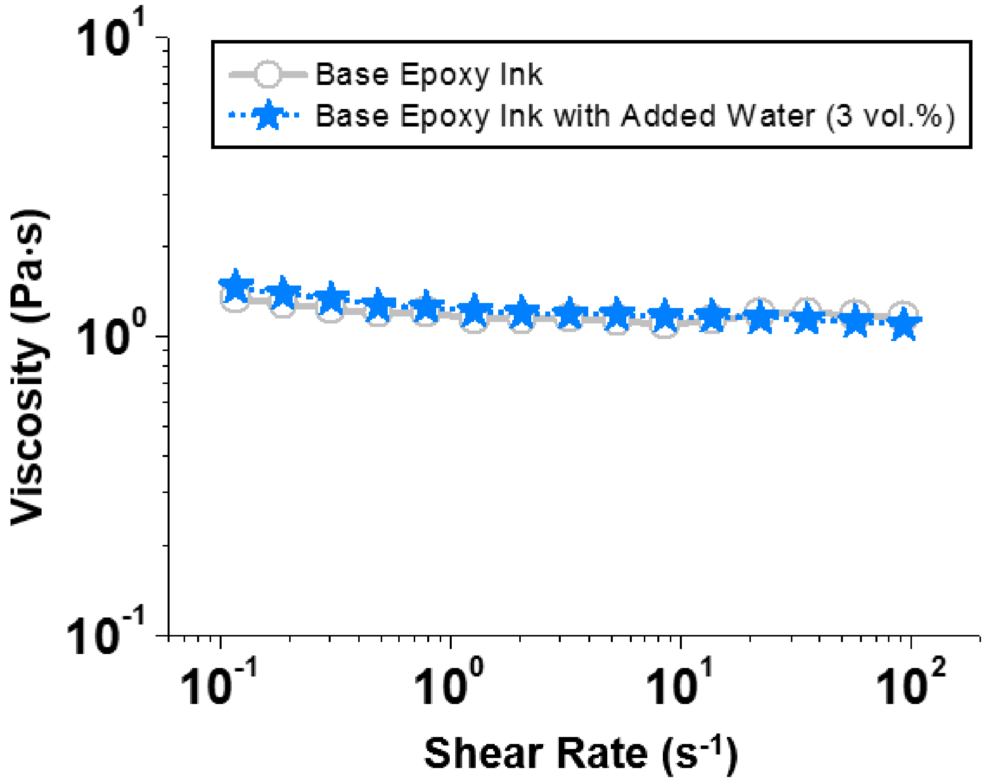

Figure S6. Viscosities of the uncured base epoxy inks without (grey squares) and with (blue stars) added water (3 vol.%), measured using rotational viscometry within the applied shear rate range between 0.1 and 100 s^{-1}.

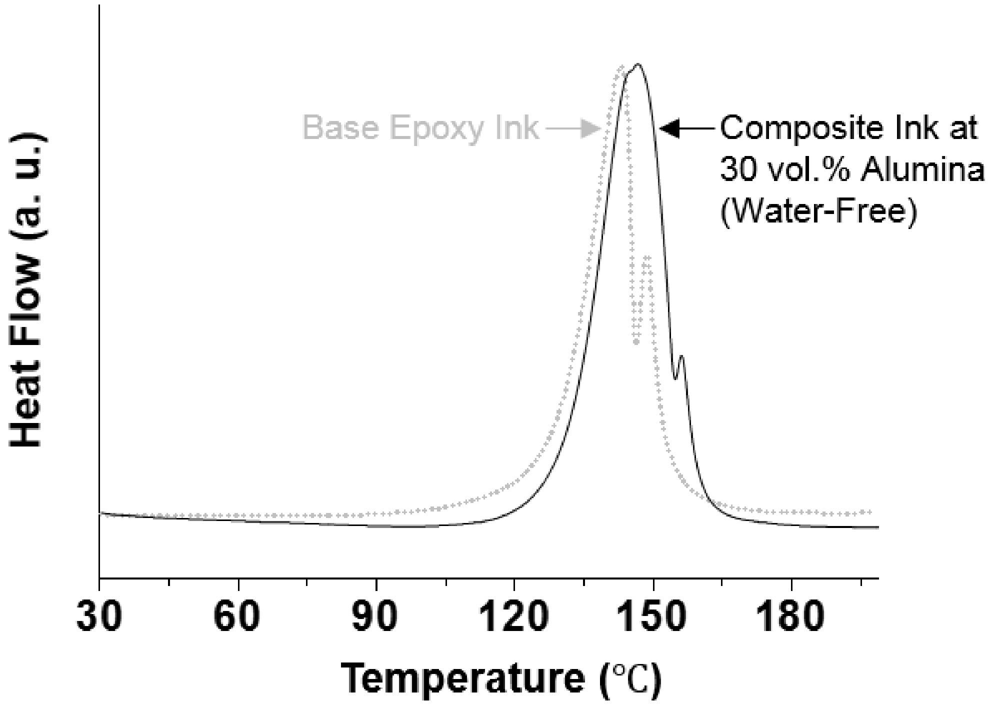

Figure S7. DSC curves for the uncured base epoxy ink (grey) and water-free epoxy/alumina ink (30 vol.% alumina).

Computer vision numerical analysis

The orientation of the particles shown in the SEM images of Figure 6 in the main text was analyzed via the computer vision (CV) technique, which was numerically expressed by the *cosine similarity* between the unit principle direction vector of the CV-detected edges of the particles and the thickness (or vertical) direction vector. This cosine similarity calculation returns the float from 0 to 1 as the direction of the particle edge goes from vertical to parallel to the thickness (or vertical) direction of the sample.

First, the raw SEM images (Figure 8a and 8f) were processed via the adaptive threshold operation to export binary images (Figure 8b and 8g) to detect the representative edges. The clusters of the linked pixels of the binary image were then segmentized via the connect components operation, and each segment was colored differently in Figure 8c and 8h. The first eigenvector for the covariance matrix of the x, y coordinates of the contour of each edge segment was then calculated, from which the principle direction vector was induced. Here, the first eigenvector is the main long axis vector to which the deviation of the contour coordinates becomes the maximum. Therefore, the first eigenvector is generally regarded as the principle direction vector of the desired coordinates (contour coordinates in this case) in the linear algebra principal axis theorem. Finally, the cosine similarity of each edge segment is calculated from the absolute value of the dot product between the unit principle direction ($\vec{v}$) and the thickness (or vertical) direction vectors ($\vec{t}$, (0, 1)).
